# Tree Biomass Allocation and Its Model Additivity for *Casuarina equisetifolia* in a Tropical Forest of Hainan Island, China

**DOI:** 10.1371/journal.pone.0151858

**Published:** 2016-03-22

**Authors:** Yang Xue, Zhongyang Yang, Xiaoyan Wang, Zhipan Lin, Dunxi Li, Shaofeng Su

**Affiliations:** Forestry Research Institute of Hainan Province, Haikou 57l100, Hainan, P. R. China; Technical University in Zvolen, SLOVAKIA

## Abstract

*Casuarina equisetifolia* is commonly planted and used in the construction of coastal shelterbelt protection in Hainan Island. Thus, it is critical to accurately estimate the tree biomass of *Casuarina equisetifolia* L. for forest managers to evaluate the biomass stock in Hainan. The data for this work consisted of 72 trees, which were divided into three age groups: young forest, middle-aged forest, and mature forest. The proportion of biomass from the trunk significantly increased with age (*P*<0.05). However, the biomass of the branch and leaf decreased, and the biomass of the root did not change. To test whether the crown radius (*CR*) can improve biomass estimates of *C*. *equisetifolia*, we introduced *CR* into the biomass models. Here, six models were used to estimate the biomass of each component, including the trunk, the branch, the leaf, and the root. In each group, we selected one model among these six models for each component. The results showed that including the *CR* greatly improved the model performance and reduced the error, especially for the young and mature forests. In addition, to ensure biomass additivity, the selected equation for each component was fitted as a system of equations using seemingly unrelated regression (SUR). The SUR method not only gave efficient and accurate estimates but also achieved the logical additivity. The results in this study provide a robust estimation of tree biomass components and total biomass over three groups of *C*. *equisetifolia*.

## Introduction

Biomass is the biological material, whereas the forest biomass, especially for tree biomass, includes all existing plant mass in the forest or arboreal fraction, including trunks, branches, leaves, and roots [1]. Due to its important carbon pool in forest ecosystems [2] and the costly and time-consuming process of collecting tree biomass in the forest, the accurate prediction of forest biomass stocks is in great need for scientists, policymakers and forest managers trying to address an increasing array of demands in forests. A number of approaches for tree biomass prediction have been reported and can be divided into three categories: (1) the direct prediction of tree biomass from the measurement of variables using allometry equations [3–6]; (2) the prediction of biomass components from biomass-volume models [7, 8]; and (3) the direct prediction of tree biomass from the biomass conversion and expansion factors (BCEF) [9–11], the biomass expansion factor (BEF) [12, 13], the root: shoot ratio [14], the wood density [15], and so on.

The direct prediction of biomass is probably the most accurate and involves felling and weighting different sizes, ages and species, calculating the biomass of tree components, and constructing the relationships between these components to tree measurement variables, namely the diameter at breast height and the total height. With the available data, a modeling study can be carried out to determine the best equation for estimating the biomass components and the total tree biomass of a given area. In most cases, modeling of biomass components and the total tree biomass are performed independently. Using these equations, the sum of the biomass components generates inconsistent results from those obtained using the total tree biomass model [1, 4], implying that the models for biomass components and the total tree biomass should be estimated together. Simultaneous estimation considering the additivity principle [16] should be used to solve the inconsistency in these estimates. Because cross-equation correlations existed among error components of the above models, a method suggested by Borders [17] was used to simultaneously estimate the parameters of the regression system. Zhang *et al*. [18] used this method to estimate the system equations of forest growth. This technique provides a statistically correlated system of equations with restrictions to parameters and ensures additivity.

*Casuarina equisetifolia*, an environmentally and economically important tree species of the *Casuarinaceae* family [19], was successfully introduced to the tropical and sub-tropical zones of China in 1897 [20–22]. *C*. *equisetifolia* has special canopy characters, such as whorls of tiny scales, fine cladodes and a tower-shaped morphological structure. These phenotypic characteristics increase wind resistance and allow for better growth in hostile coastal environments [23]. In the green shelterbelts in southern China, *C*. *equisetifolia* is one of the key species for coastal protection against typhoons and tsunamis, as well as for wood and fuel wood production. *C*. *equisetifolia* was introduced to Hainan Island, China, in the 1950s and immediately became the dominant species due to its pioneer characteristics, including fast growth, adaptability to degraded sites for soil improvement, and ability to resist wind [23]. Under global climate change and multifunctional forest management, it is critical to accurately estimate the tree biomass of *C*. *equisetifolia* for forest managers and policymakers evaluating the carbon stock in Hainan Island. Wu *et al*. [24] established root biomass equations of *C*. *equisetifolia* clones in northeastern Hainan Island. Yang *et al*. [20] developed tree biomass models of *C*. *equisetifolia* in Hainan. However, the biomass models were not developed by age groups. There were significant differences in the biomass concentrations among the different parts of the tree at different age groups [25, 26]. In addition, the biomass models that were developed by Yang *et al*. [27] did not account for the variable crown radius (*CR*). The tree *CR* is considered an indirect measure of the photosynthetic capacity of trees [28]. Goodman *et al*. [29] found that the measurement of *CR* was critical to improving the estimation of tree biomass.

The objective of this study was (1) to develop a tree component biomass model and a total biomass model of *C*. *equisetifolia* by age groups in the tropical forest of Hainan Island; (2) to test the importance and influence of *CR* on the estimation of biomass components; and (3) to ensure the additivity of tree biomass components.

## Study Sites

This study was conducted in the northeast coastal zone of Hainan Island (E: 110°36′-111°01′,N: 19°40′-20°06′), adjacent to the South China Sea in the monsoon tropics of south China. The elevation ranges from 5 m to 70 m above sea level, and the highest elevation is 70 m. The tropical marine climate ensures an annual rainfall of 1721.6 mm. The mean annual air temperature varies between 23.4°C and 24.4°C, and the minimum and maximum temperatures are 17°C and 36°C, respectively. The soil structure was loose, with good permeability but low water-retention properties. Most of the tree species in the study sites are *C*. *equisetifolia*, *Pinus elliottii*, *Acacia mangium*, *Acacia auriculiformis*, *Acacia crassicarpa*, *Pinus caribaea*, and *Calophyllum inophyllum*. Among these tree species, *C*. *equisetifolia* is the dominant tree species, accounting for 79% of these species.

## Methods

### Data collection

The data used in this study were from a systematic sampling of permanent, square-shaped plots (0.067 ha) across northeast Hainan Island that were aggregated over a 4×3 km grid. A total of 72 *C*. *equisetifolia* trees were randomly collected from these plots and divided into three groups [22]: young (age≤5yrs), middle-aged (6<age≤15yrs) and mature (15yrs<age). The plantations were authorized by the Forestry Research Institute of Hainan Province. The field studies did not involve endangered or protected species.

The tree biomass was measured using the destructive method. The crown width was measured in two directions at 90° angles from each other and averaged before the tree was felled. After the tree was felled, the diameter at breast height (*D*) and the total tree height (*H*) were measured in the field. The fresh biomass was obtained by weighing the four components of each tree separately: the trunk, the branch, the leaf, and the root. The trunk was cut into 3 segments and weighed separately, considering the different moisture contents in the whole trunk, and three subsamples (approximately 500 g each) of each segment were selected and weighed in the field. In addition, three subsamples of the branches and leaves (approximately 500 g each) were selected and weighed in the field and transported back to the laboratory for drying. In terms of roots, the root system is often partially removed from the soil [30,31]. However, the disadvantage of sampling procedure is that the observed root biomass is less accurately determined compared to excavating in full [32]. Here, the whole roots of all of the sample trees were excavated. A trench was dug to the extent of the crown coverage, and the depth of excavation depended on the depth of the taproot. The fresh weights of the stump, the coarse roots (more than 10 mm), and the small roots (2–10 mm, not including fine roots less than 2 mm) were measured. In addition, subsamples were selected and weighed in the field and transported to the laboratory.

After being transported to the laboratory, the subsamples were oven-dried at 85°C until a constant weight was obtained. Dry weights were computed for all tree components using the ratio of dry weight to fresh weight from subsamples from each component and multiplied by their known fresh weights. The total tree biomass was generated by summing the biomass of each component. The statistics of the tree variables and the biomass of each component at different age groups are listed in Table 1.

**Table 1:** Tree variables and biomass of each component of *C*. *equisetifolia* in different age groups.

Age group		*D*/cm	*H*/m	*CR*/m	*W*_*T*_/kg	*W*_*B*_/kg	*W*_*L*_/kg	*W*_*R*_/kg
Young(*n* = 18)	Mean	6.317	9.183	3.332	12.388	2.593	2.958	5.008
	SD.	2.759	3.359	0.840	14.861	2.111	2.773	5.669
	Max.	13.1	15.4	5.725	58.959	8.163	12.07	20.751
	Min.	2.5	4.1	1.95	0.664	0.326	0.627	0.515
Middle(*n* = 19)	Mean	12.747	14.016	3.834	52.450	10.534	7.315	19.885
	SD.	2.132	2.869	0.644	23.788	6.853	3.308	9.832
	Max.	16.8	19.5	5.05	103.707	34.249	15.258	40.798
	Min.	9.3	8	2.85	18.243	3.871	2.569	7.343
Mature (*n* = 35)	Mean	23.12	17.589	6.166	237.858	37.271	26.427	88.173
	SD.	5.092	2.207	1.876	111.181	32.687	31.652	61.748
	Max.	36.3	22.7	10.55	537.391	155.955	153.917	291.325
	Min.	15.6	14.1	3.4	91.731	11.208	7.618	29.106

Note: *D* is the diameter at breast height; *H* is the total tree height; *CR* is the crown radius; *W*_*t*_ is the biomass of the trunk; *W*_*B*_ is the biomass of the branch; *W*_*L*_ is the biomass of the leaf; *W*_*R*_ is the biomass of the root; SD is the standard deviation.

### Tree biomass model

In this study, the direct prediction of the tree biomass from measurement variables was used to estimate the tree biomass of *C*. *equisetifolia* in the tropical forest of Hainan Island. The standard tree biomass equation predicts tree biomass as a power function of the diameter at breast height [33]. However, in some cases, other variables, such as the total tree height and the crown radius, are also important predictors [34–36]. These equations have biologically meaningful coefficients related to the theory of “allometric” scaling relationships [37]. Niklas [38] demonstrated that the allometric relationships changed, even within the lifetime of individuals of a single species. Here, we used the following six equations to estimate the tree component biomass according to three age groups, including young, middle-aged, and mature forests.

$$W=\alpha D^{b}$$ (1)

$$W=\alpha D^{b}H^{c}$$ (2)

$$W=\alpha D^{b}CR^{c}$$ (3)

$$W=\alpha {\left(D^{2}H\right)}^{b}$$ (4)

$$W=\alpha {\left(D^{2}H\right)}^{b}CR^{c}$$ (5)

$$W=\alpha D^{b}H^{c}CR^{d}$$ (6)

where *W* is tree biomass in kg, and *α*, *b*, *c*, and *d* are the parameters to be estimated. Overman *et al*. [39] reported that it is convenient to use logarithms for the fitting model and for dealing with heteroscedasticity. Therefore, Eqs (1) to (6) can be linearized using logarithms in the following equations, respectively:

ln(W)=a+bln(D) (7)

ln(W)=a+bln(D)+cln(H) (8)

ln(W)=a+bln(D)+cln(CR) (9)

$$\text{ln}(W)=a+b\text{ln}(D^{2}H)$$ (10)

$$\text{ln}(W)=a+b\text{ln}(D^{2}H)+c\text{ln}(CR)$$ (11)

ln(W)=a+bln(D)+cln(H)+dln(CR) (12)

where *a* = ln(*α*). The log-transformation of the data leads to a biased biomass estimation [40, 41], and uncorrected biomass estimates are theoretically expected to generate a systematic underestimation. The bias is not an arithmetic constant value but rather a constant proportion of the estimation [42]. Baskerville [43] recognized this detail and gave a multiplicative correction factor for this bias:

$$CF=\text{exp}(\frac{\sigma^{2}}{2})$$ (13)

where $\sigma^{2}=\sum {\left(\text{ln}W-\text{ln}\hat{W}\right)}^{2}/(n-2)$ is the mean square error from the logarithmic regression, and *n* is the sample size. Therefore, the estimated tree biomass ($\hat{W}$) could be calculated from Eq (14):

$$\hat{W}=\text{exp}(\hat{\text{ln}W})CF$$ (14)

### Model selection

The coefficient of determination (R^2^) is the most widely used criterion in biomass model literature. However, in many situations, it has been used uncritically. The R^2^ is deceptive because it increases with the addition of polynomial terms and with the inclusion of new predictors [44, 45]. Therefore, R^2^ can sometimes be a poor estimator of model performance. The mean absolute prediction error (MAPE) [33, 46, 47] was used as the primary metric to evaluate and compare the performance of models, whose statistical properties are well known and commonly used in forecasting and model comparison in ecology and environment assessment.

$${\text{R}}^{2}=1-\Sigma {\left(W_{i}-{\hat{W}}_{i}\right)}^{2}/\Sigma {\left(W_{i}-\bar{W}\right)}^{2}$$ (15)

$$\text{MAPE}=\frac{1}{n}\sum_{i=1}^{n}\frac{\left|W_{i}-{\hat{W}}_{i}\right|}{W_{i}}$$ (16)

where *W*_*i*_ = observed biomass value tree *i*, and ${\hat{W}}_{i}$ and $\bar{W}$ = the estimated value and the average, respectively, of *W*_*i*_.

In this study, we first used the above Eqs (7–12) and Eq (14) to estimate the biomass of tree components, including the trunk, the branch, the leaf and the root, by age groups. Then, we selected the best equation according to the evaluation statistics (R^2^ and MAPE) and the significance of the estimated parameters. Finally, the best equation for each component was fitted as a system of equations ensuring *W*_*Total*_ = *W*_*T*_ + *W*_*B*_ + *W*_*L*_ + *W*_*R*_ using a seemingly unrelated regression (SUR) [48]:

$$\left\{ \begin{array}{l} \text{ln}(W_{T})=f(D,H,CR)\\ \text{ln}(W_{B})=f(D,H,CR)\\ \text{ln}(W_{L})=f(D,H,CR)\\ \text{ln}(W_{R})=f(D,H,CR)\\ W_{Total}=\text{exp}(\text{ln}W_{T})CF_{T}+\text{exp}(\text{ln}W_{B})CF_{B}+\text{exp}(\text{ln}W_{L})CF_{L}+\text{exp}(\text{ln}W_{R})CF_{R}) \end{array}\right.$$ (17)

where *CF*_*T*_, *CF*_*B*_, *CF*_*L*_, *CF*_*R*_ are the correction factors for trunk biomass, branch biomass, leaf biomass, and root biomass by age groups, respectively. The fitting procedure involved the use of option SUR of the procedure MODEL in SAS. The normality of the residual for total biomass prediction (${\hat{W}}_{Total}$) was tested using the normal Q-Q plot and the Shapiro-Wilk test of normality.

## Results

### Biomass allocation

**Fig 1:**
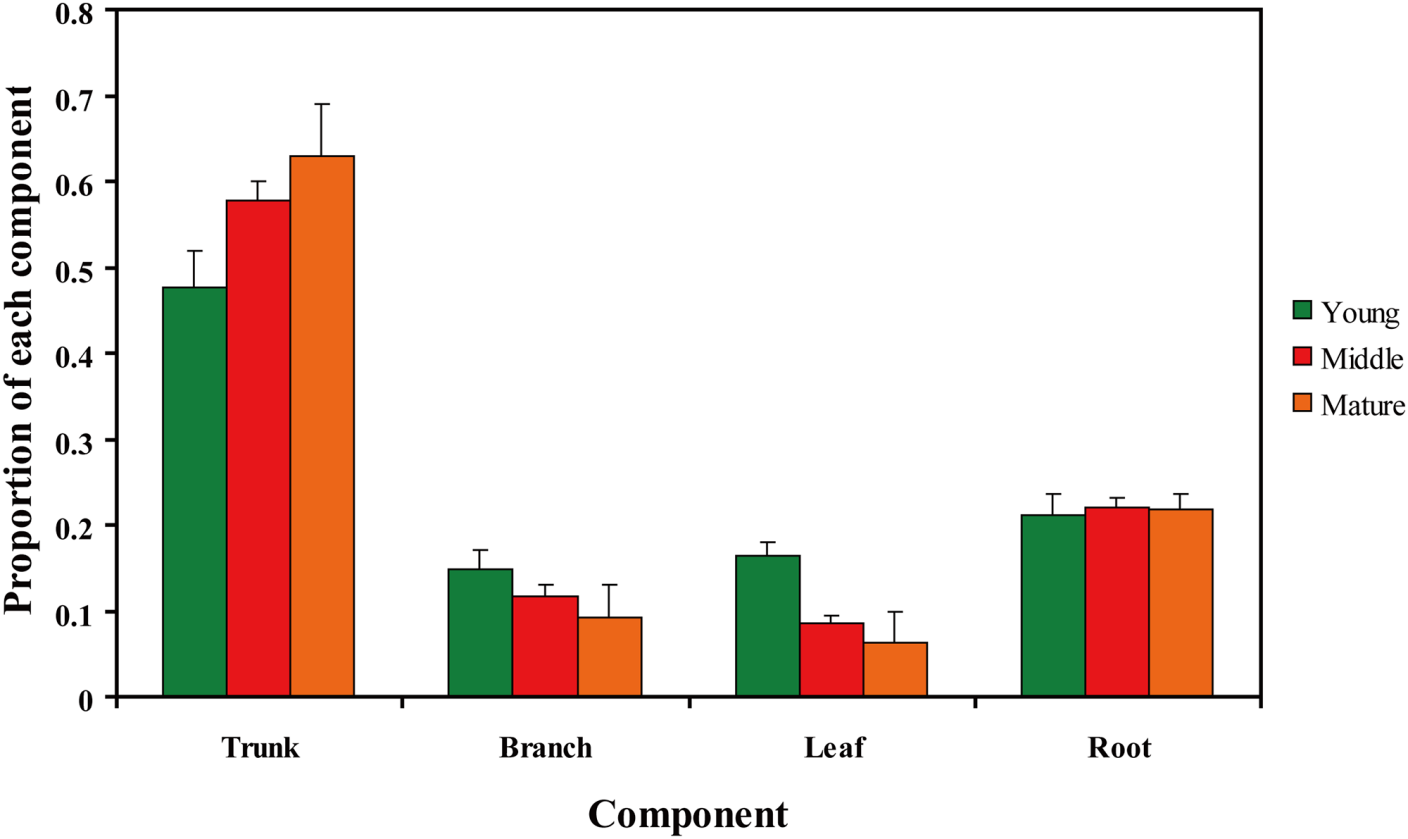
Proportion of the tree biomass from the trunk, the branch, the leaf, and the root in young, middle-aged, and mature forests.

The tree biomass of *C*. *equisetifolia* over the three age groups was highest in the trunk, followed by (in decreasing order) the root, the branch, and the leaf. The proportion of the biomass from trunk increased with forest age, while that in the branch and the leaf declined, especially for the leaf, which significantly declined from the young forest to the middle-aged forest (ANOVA analysis, *F* = 30.457, *P* <0.001). In addition, the proportion of biomass of *C*. *equisetifolia* that from the root was independent of tree age (ANOVA analysis, *F* = 0.276, *P* = 0.76). Of the total biomass, the trunk accounted for 47.6% to 62.9% from the young forest to the mature forest, respectively, the branch for 14.9% to 9.2%, the leaf for 16.4% to 6.2%, and the root for 21.1% to 21.7% (S1 Table, Fig 1).

### Each component biomass model selection by age groups

We developed the tree component biomass models for young, middle-aged and mature forests using Eqs (7–12), and the correction factor was applied to back-transformed predictions (Eq 14). The R^2^ and the MAPE were calculated based on the back-transformed predictions.

In the young forest, both Eqs 8 and 10 performed the best in estimating the trunk biomass compared to other models in terms of the MAPE, the R^2^, and the parameter significance. Both of these equations explained 99.4% of the variance in the trunk biomass, and the MAPE was 0.071 (Table 2). Here we used the Eq 8 to model the trunk biomass. The best model for estimating the branch biomass was Eq 9 compared to other models in terms of the MAPE and parameter significance. The second performance model Eq 11 resulted in a 1.26% increase in the MAPE over the best model (Eq 9). The best model introducing the variable *CR* explained 85.3% of the variance in the branch biomass. Although the MAPE in Eq 12 was equal to Eq 9, the two-parameter estimates of this model were not significant at a level of 0.05. In terms of the MAPE and the parameter estimate significance, the best model for estimating the leaf biomass was Eq 9, in which the MAPE was 0.341, lower than that of the other models. For the root biomass model, Eq 11 with the variables *D*^2^*H* and *CR* was the best model, as the MAPE was the lowest, and the R^2^ was the highest. In addition, the three parameters of this model were all significant at a level of 0.05.

**Table 2:** Parameter estimates and model evaluation statistics of each biomass model for the young forest.

Component	model	*a*	*B*	*c*	*d*	R^*2*^	MAPE
	Eq (7)						
Trunk		-2.785*	2.678*	-	-	0.994	0.110
Branch		-1.776*	1.407*	-	-	0.820	0.391
Leaf		-1.702*	1.422*	-	-	0.795	0.379
Root		-2.841*	2.259*	-	-	0.876	0.175
	Eq (8)						
Trunk		-3.413*	1.884*	0.941*	-	**0.994**	**0.071**
Branch		-1.322*	1.980*	-0.680	-	0.843	0.372
Leaf		-1.272*	1.965*	-0.644	-	**0.829**	0.367
Root		-3.110*	1.919*	0.403	-	0.878	0.168
	Eq (9)						
Trunk		-2.637*	2.757*	-0.244	-	0.991	0.103
Branch		-2.466*	1.041*	1.134*	-	0.853	**0.315**
Leaf		-2.208*	1.154*	0.831*	-	0.746	**0.341**
Root		-3.094*	2.125*	0.416*	-	0.933	0.159
	Eq (10)						
Trunk		-3.414*	0.942*	-	-	**0.994**	**0.071**
Branch		-2.022*	0.480*	-	-	0.773	0.436
Leaf		-1.954	0.485*	-	-	0.742	0.409
Root		-3.343*	0.789*	-	-	0.874	0.163
	Eq (11)						
Trunk		-3.414*	0.942*	0.001	-	0.994	0.071
Branch		-2.743*	0.349*	1.245*	-	0.826	0.319
Leaf		-2.510*	0.384*	0.960*	-	0.703	0.348
Root		-3.693*	0.726*	0.604*	-	**0.952**	**0.134**
	Eq (12)						
Trunk		-3.415*	1.883*	0.942*	0.001	0.994	0.071
Branch		-2.388*	1.128	-0.094	1.109*	**0.858**	0.315
Leaf		-2.011*	1.375*	-0.239	0.769	0.764	0.348
Root		-3.689*	1.456*	0.721*	0.603*	0.950	0.135

Note: Values in bold denote the best statistic among six biomass models for each component.

* means significant at 0.05 level.

In the middle-aged forest, Eq 8 explained 92.4% of the variance in the trunk biomass (Table 3). In addition, the MAPE was lowest, 55.2% lower than that of the equation with only *D* (Eq 7), indicating that the total tree height greatly improved the trunk biomass model. For the branch biomass model, although the MAPE from Eq 12 was the lowest, the parameters of *H* and *CR* were not significant at a level of 0.05. Eq 7 was the best for estimating the branch biomass and the leaf biomass. In terms of the root biomass, both the MAPE and the R^2^ showed that Eq 10 was the best model.

**Table 3:** Parameter estimates and model evaluation statistics of each biomass model for the middle-aged forest.

Component	model	*a*	*b*	*c*	*d*	R^2^	MAPE
	Eq (7)						
Trunk		-2.108*	2.354*	-	-	0.776	0.203
Branch		-4.222*	2.538*	-	-	0.529	0.268
Leaf		-3.164*	1.996*	-	-	0.587	0.288
Root		-3.018*	2.326*	-	-	0.627	0.277
	Eq (8)						
Trunk		-2.901*	1.103*	1.513*	-	**0.924**	**0.091**
Branch		-3.977*	2.925*	-0.467	-	**0.537**	0.266
Leaf		-2.8199	2.541*	-0.660	-	0.580	0.284
Root		-3.337*	1.822*	0.609	-	0.696	0.264
	Eq (9)						
Trunk		-2.637*	2.104*	0.872*	-	0.793	0.198
Branch		-4.185*	2.556*	-0.061	-	0.532	0.269
Leaf		-3.139*	2.008*	-0.042	-	0.583	0.289
Root		-3.308*	2.189*	0.479	-	0.642	0.258
	Eq (10)						
Trunk		-3.157*	0.912*	-	-	0.923	0.114
Branch		-3.463*	0.738*	-	-	0.413	0.311
Leaf		-2.305*	0.546*	-	-	0.438	0.346
Root		-3.257*	0.798*	-	-	**0.706**	**0.252**
	Eq (11)						
Trunk		-3.217*	0.869*	0.294	-	0.908	0.124
Branch		-3.382*	0.797*	-0.404	-	0.435	0.316
Leaf		-2.254*	0.583*	-0.251	-	0.429	0.347
Root		-3.263*	1.793*	0.032	-	0.706	0.265
	Eq (12)						
Trunk		-2.888*	1.098*	1.532*	-0.038	0.924	0.091
Branch		-4.083*	2.961*	-0.618	-0.306	0.524	**0.264**
Leaf		-2.991*	2.601*	-0.902	0.495	**0.597**	0.275
Root		-3.395*	1.842*	0.528	0.165	0.696	0.260

Note: Values in bold denote the best statistic among six biomass models for each component.

* means significant at 0.05 level.

In mature forests, Eq 7 with only *D* was best in terms of having the lowest MAPE and the second highest R^2^ (Table 4). Although the MAPE values from Eqs 7 and 8 were equal, the effect of *H* on the trunk biomass was not significant for this dataset (*P*>0.05). The best equation for estimating the branch biomass was Eq 10 compared to other models in terms of the MAPE and the R^2^. Eq 9 with variables *D* and *CR* had the lowest MAPE compared to the other models in estimating the biomass of the leaf and the root. In terms of leaf biomass, adding *CR* greatly improved estimates, increasing the R^2^ and reducing the MAPE compared to the equivalent equation without *CR* (Eq 7).

**Table 4:** Parameter estimates and model evaluation statistics of each biomass model for the mature forest.

Component	model	*a*	*b*	*c*	*d*	R^2^	MAPE
	Eq (7)						
Trunk		-0.963*	2.032*	-	-	0.936	**0.105**
Branch		-3.945*	2.349*	-	-	0.681	0.394
Leaf		-4.108*	2.270*	-	-	0.488	0.399
Root		-3.566*	2.525*	-	-	0.909	0.132
	Eq (8)						
Trunk		-0.890*	2.050*	-0.044	-	0.936	0.105
Branch		-4.297*	2.262*	0.218	-	0.689	0.394
Leaf		-4.652*	2.136*	0.337	-	0.491	0.398
Root		-3.379*	2.571*	-0.116	-	0.910	0.132
	Eq (9)						
Trunk		-0.890*	1.985*	0.042	-	**0.937**	0.106
Branch		-4.159*	2.488*	-0.124	-	0.681	0.396
Leaf		-3.187*	1.672*	0.532*	-	0.503	**0.380**
Root		-3.170*	2.268*	0.228*	-	0.921	**0.126**
	Eq (10)						
Trunk		-2.081*	0.819*	-	-	0.909	0.116
Branch		-5.313*	0.956*	-	-	**0.692**	**0.384**
Leaf		-5.466*	0.927*	-	-	0.483	0.406
Root		-4.938*	1.016*	-	-	0.868	0.154
	Eq (11)						
Trunk		-1.804*	0.767*	0.116	-	0.914	0.116
Branch		-5.467*	0.985*	-0.064	-	0.691	0.385
Leaf		-4.129*	0.671*	0.559*	-	0.502	0.385
Root		-4.165*	0.868*	0.323*	-	0.893	0.153
	Eq (12)						
Trunk		-0.806*	2.004*	-0.050	0.043	0.936	0.106
Branch		-4.551*	2.403*	0.235	-0.131	0.689	0.395
Leaf		-3.641	1.573*	0.271	0.523*	**0.506**	0.380
Root		-2.928*	2.320*	-0.145	0.233*	**0.922**	0.126

Note: Values in bold denote the best statistic among six biomass models for each component.

* means significant at 0.05 level.

### Biomass predictions using SUR method by age groups

Based on the best model for each component analyzed above, we used SUR method to estimate a system of equations (Eq (15)) to ensure the additivity of the total tree biomass, including the trunk biomass, the branch biomass, the leaf biomass, and the root biomass.

**Fig 2:**
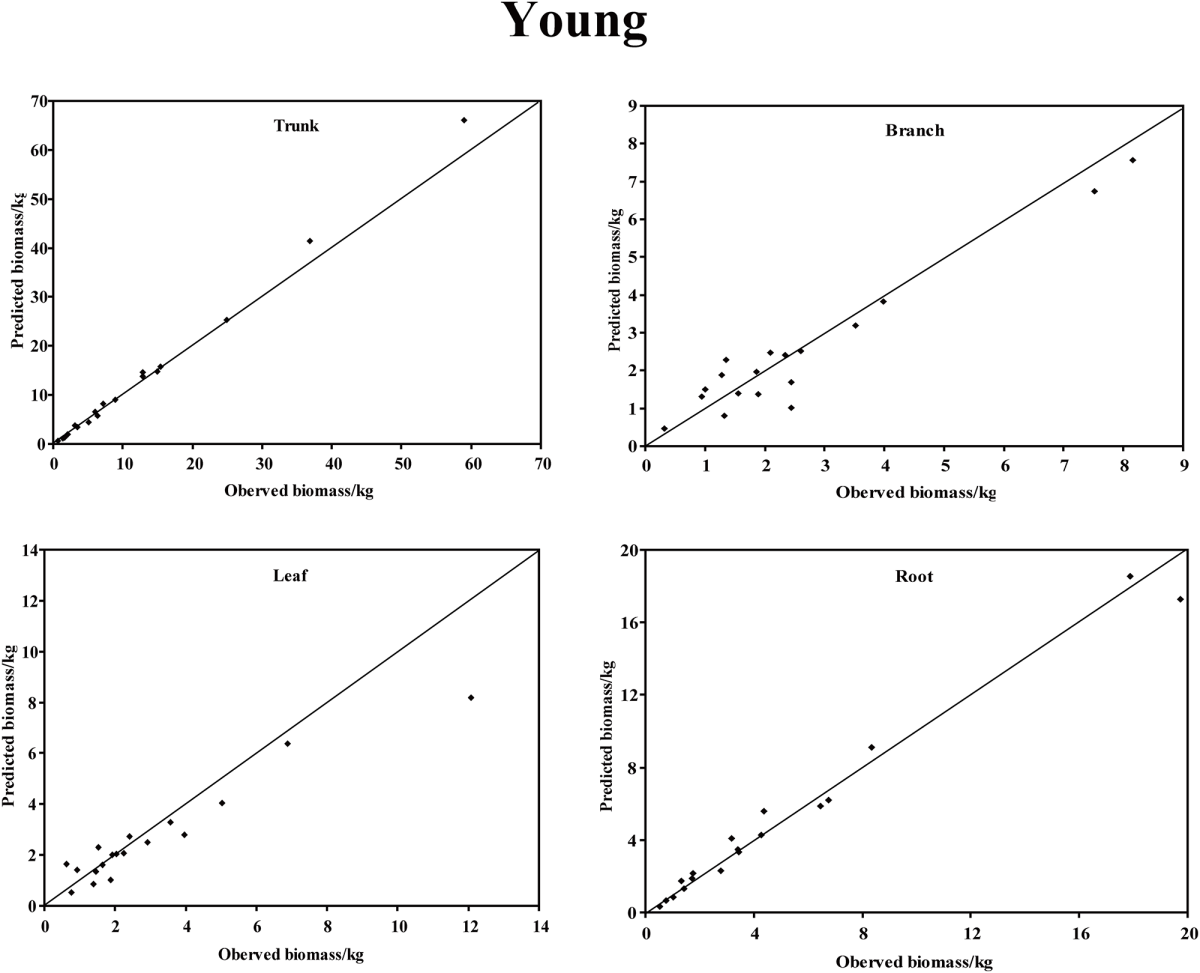
The relationship between the predicted biomass and observed biomass for four components in young forest.

**Fig 3:**
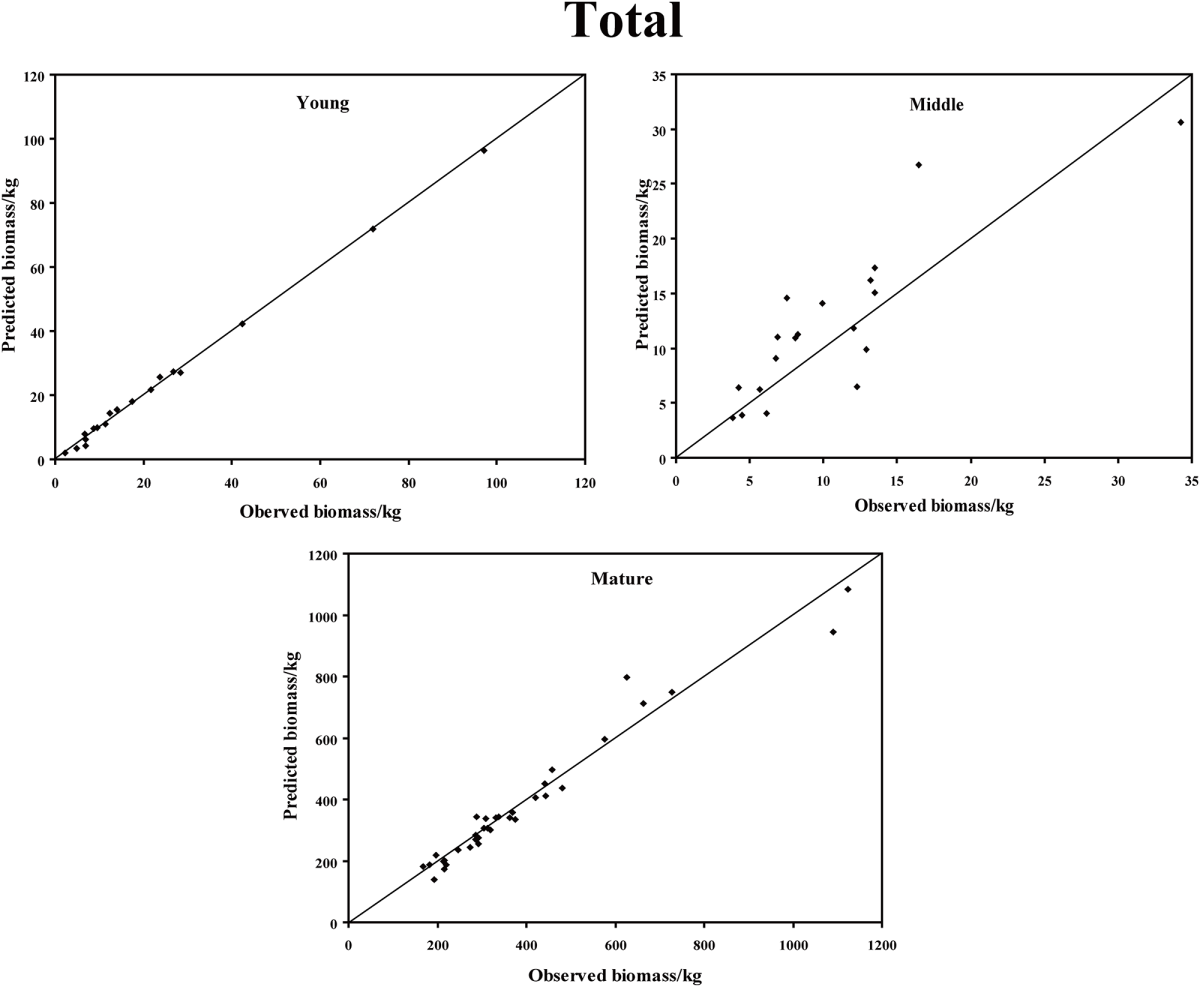
The relationship between the predicted biomass and observed biomass for total biomass in young, middle-aged, and mature forests.

**Fig 4:**
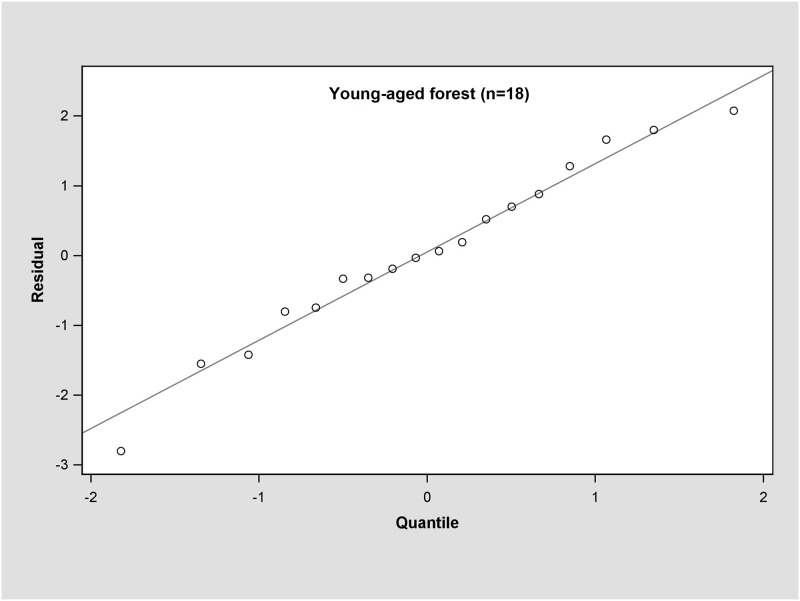
Q-Q plot of the total biomass estimation for young forest.

**Fig 5:**
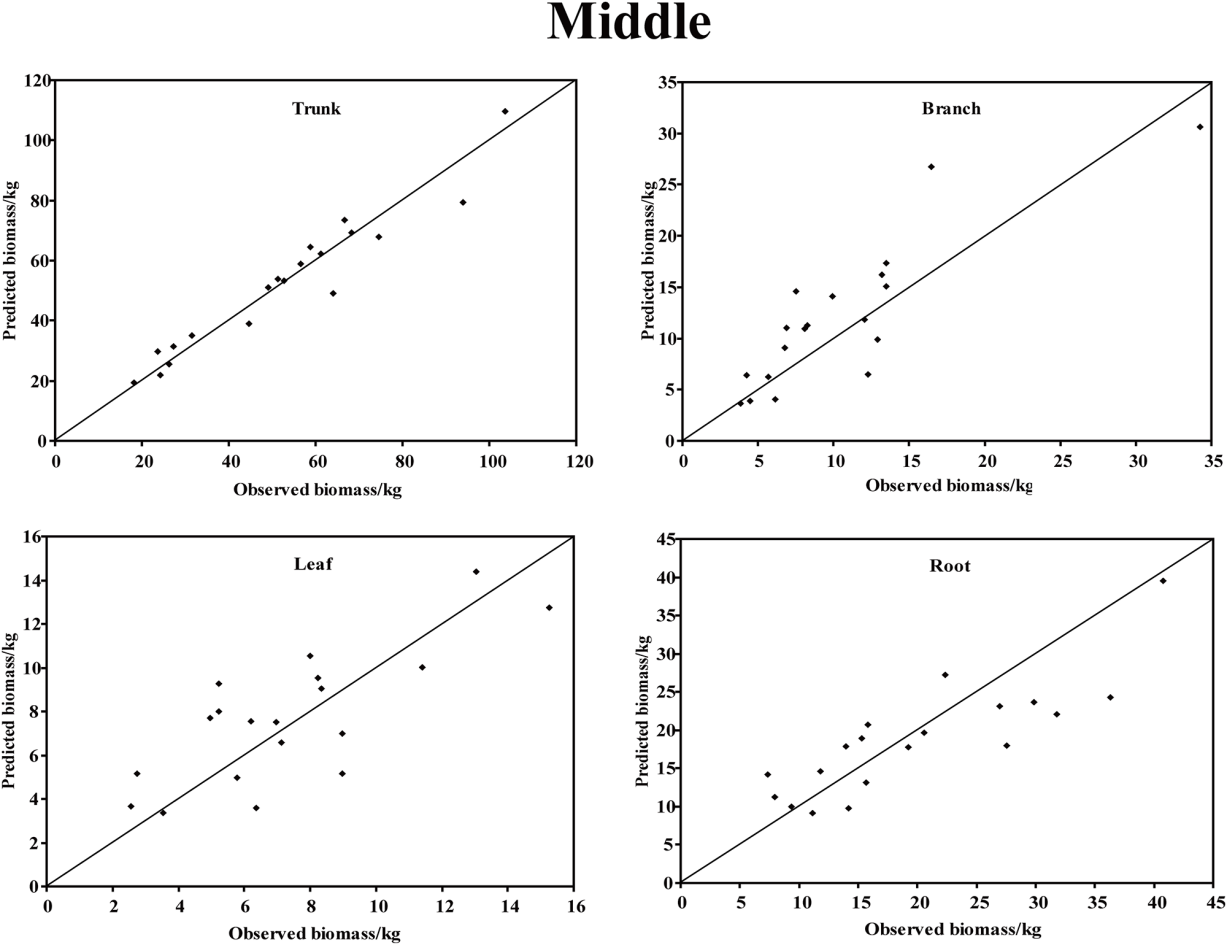
The relationship between the predicted biomass and observed biomass for four components in middle-aged forest.

**Fig 6:**
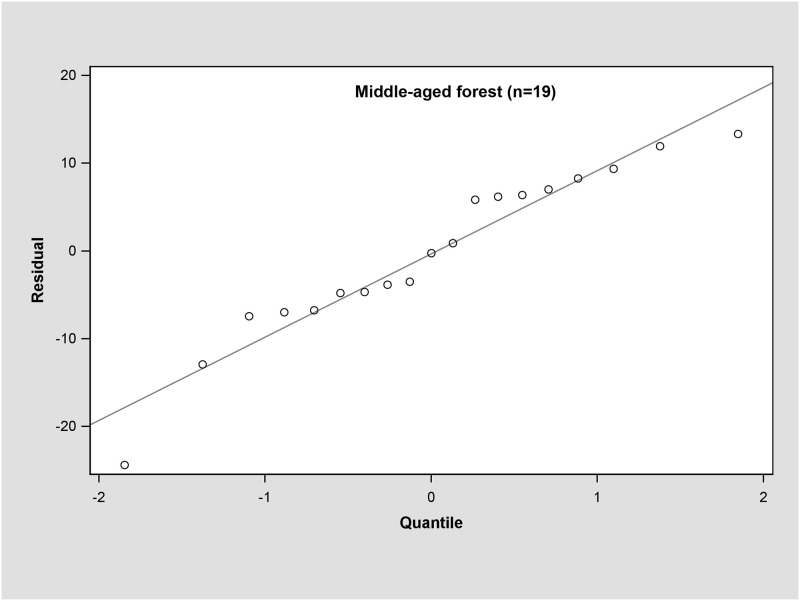
Q-Q plot of total biomass estimation for middle-aged forest.

**Fig 7:**
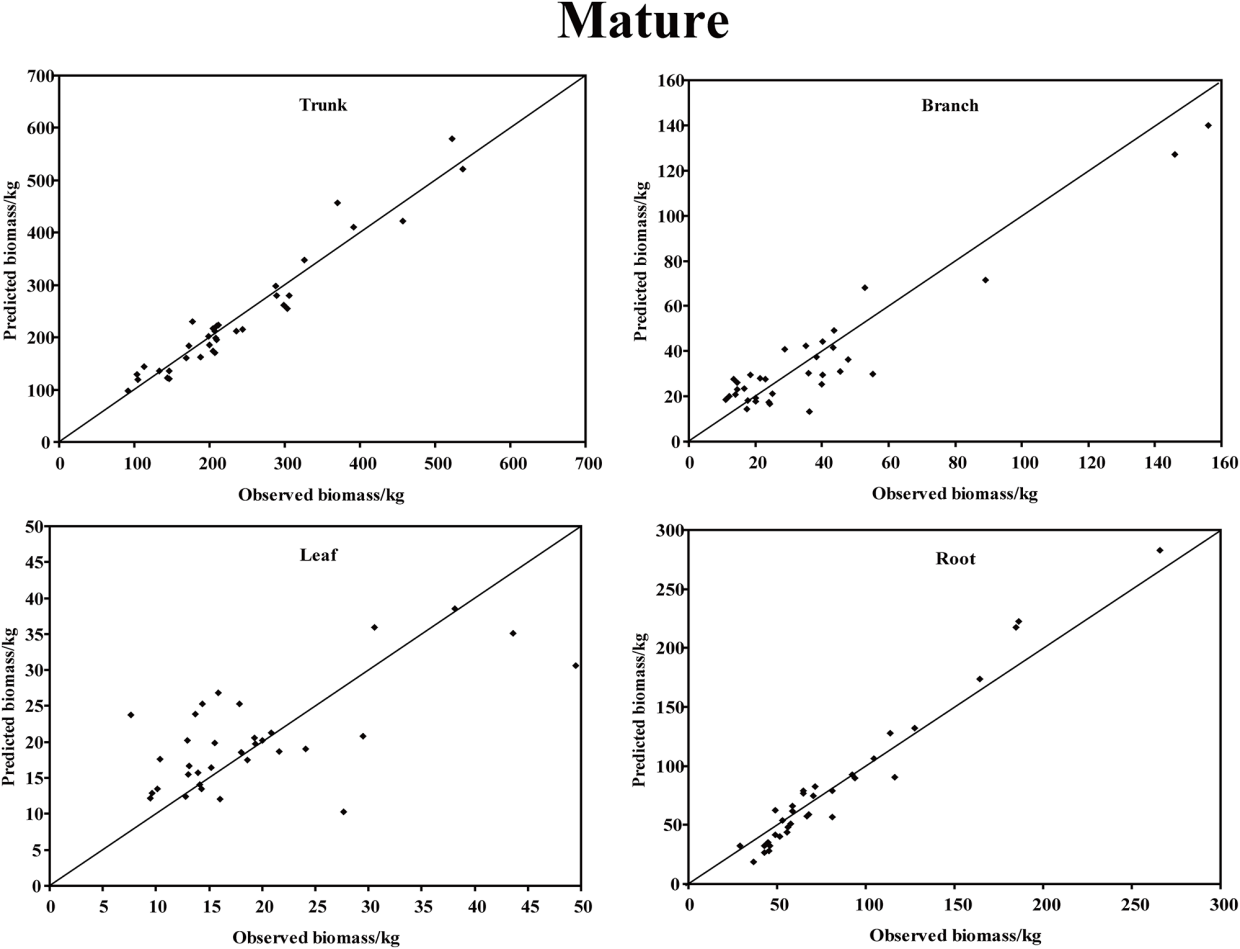
The relationship between the predicted biomass and observed biomass for four components in mature forest.

**Fig 8:**
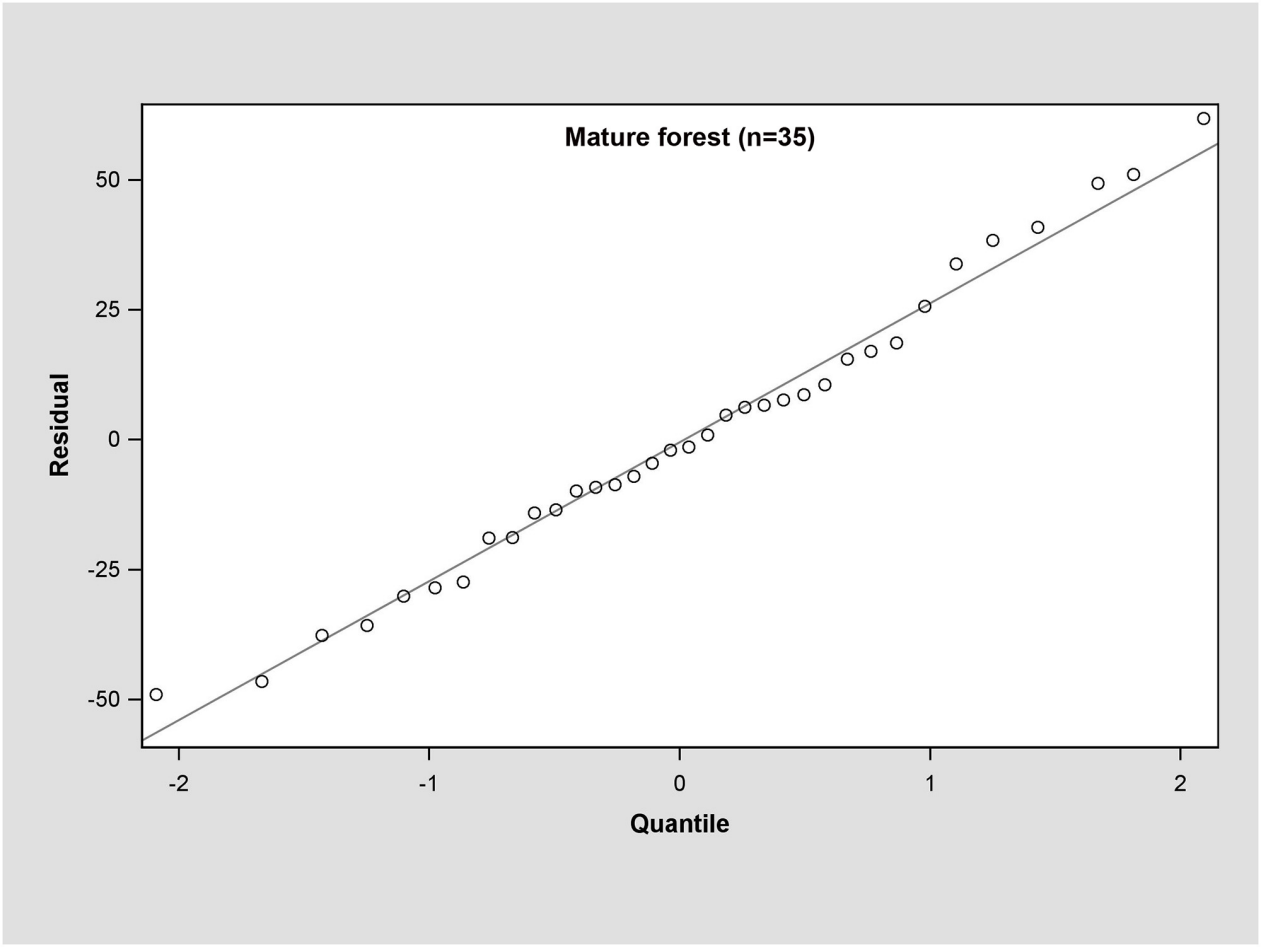
Q-Q plot of the total biomass estimation for the mature forest.

In the young forest, the trunk biomass predictions were close to the observed biomass, as well as the branch biomass, leaf biomass and root biomass (Fig 2). In terms of the total biomass predictions, the predictions were highly correlated with the observed values (Fig 3), and the residual was normal (Fig 4, Shapiro-Wilk test, *P* = 0.891>0.05). In the middle-aged forest, the fit accuracy of the trunk biomass was very high (Fig 5). However, the branch biomass, leaf biomass and root biomass prediction performed worse than the trunk biomass (Fig 5). In addition, the total biomass predictions were very close to observed values (Fig 3), and the residual for total biomass predictions was also normal (Fig 6, Shapiro-Wilk test, *P* = 0.274>0.05). The SUR method also showed good performance on predicting the trunk biomass, branch biomass, leaf biomass, root biomass in the mature forest (Fig 7), as well as the total biomass which could be depicted by the relationship and residual plots (Shapiro-Wilk test, *P* = 0.839>0.05, Figs 3 and 8). All the parameter estimates of the selected biomass models by age groups using SUR method are displayed in Table 5.

**Table 5:** Parameter estimates of the selected models for different components by age groups using SUR method.

Component	Age groups	*a*	*b*	*c*
	Young			
Trunk		-3.599	2.274	0.709
Branch		-2.436	1.123	0.953
Leaf		-2.158	1.211	0.640
Root		-3.983	0.771	0.625
	Middle			
Trunk		-2.822	1.218	1.375
Branch		-6.197	3.358	-
Leaf		-4.247	2.450	-
Root		-2.620	0.712	-
	Mature			
Trunk		-1.211	2.109	-
Branch		-5.127	0.935	-
Leaf		-2.704	1.827	-
Root		-5.487	2.820	0.534

Note: In young forest, trunk: ln(*W*) = *a* + *b*ln(*D*) + *c*ln(*H*); branch and leaf: ln(*W*) = *a* + *b*ln(*D*) + *c*ln(*CR*); root: ln(*W*) = *a* + *b*ln(*D*^2^*H*) + *c*ln(*CR*). In middle-aged forest, trunk: ln(*W*) = *a* + *b*ln(*D*) + *c*ln(*H*); branch and leaf: ln(*W*) = *a* + *b*ln(*D*); root: ln(*W*) = *a* + *b*ln(*D*^2^*H*). In mature forest, trunk and leaf: ln(*W*) = *a* + *b*ln(*D*); branch: ln(*W*) = *a* + *b*ln(*D*^2^*H*); root: ln(*W*) = *a* + *b*ln(*D*) + *c*ln(*CR*).

## Discussion

Variation in the tree biomass and its allocation to components was commonly found in comparisons among individuals, ages, stands, regions, and species [49–51]. In the study, the proportion of trunk biomass to total tree biomass significantly increased at a level of 0.05 in the young forest to the mature forest and represented the greatest portion of the total biomass, which also could be found in other studies [52–53]. However, the proportion of branch biomass and leaf biomass decreased from the young forest to the mature forest. The result is consistent with findings of Scots pine (*Pinus sylvestris*) [54] and loblolly pine (*Pinus taeda*) [55]. The leaf biomass is a valuable component to quantify because it is highly correlated with forest productivity in young forests, which typically peak as canopies close and then decreases with stand age [56–57]. The relative amount of biomass from the leaf in this study significantly decreased from the young forest to the middle-aged forest (Fig 1). The individual-tree root biomass increases with tree age to maintain a balance between the above- and belowground components [58–59]. Although the root biomass increased with tree age, the proportion of root biomass to the total tree remained stable at the three stages, indicating that roots are a crucial component when considering biomass partitioning for *C*. *equisetifolia*. Bijak *et al*. [60] reported a decrease in the proportion of root biomass in relation to total biomass of the silver birch (*Betula pendula*) with the increasing tree age, which was different in this study. *C*. *equisetifolia* has a strong root system to be adaptable to degraded sites, which results in a high proportion of biomass allocation at the whole stand development stages.

The main predictor of biomass, *D*, tends to work quite well for predicting tree biomass [61–63], but it fails to provide accurate estimates of biomass component fractions [64]. Many studies have shown that improvements can be made by adding variables other then *D* to improve tree biomass estimation. The most widely used variable is the total tree height because height-diameter relationships vary across a range of ecological conditions [65–67]. Chave et al. [68] found that the inclusion of height reduced the standard error of aboveground biomass estimates from 19.2 to 12.5% in predicting the biomass of tropical forests. As noted above, more than 50% of the aboveground biomass was from the trunk. In the trunk biomass models of this study, the MAPE from equation with variable *H* was 35.45% and 55.17% lower than that of the equation with only the variable *D* in the young and middle-aged forests, respectively. In the leaf biomass equations, other variables, such as tree age, crown competition factors [69] that are closely related to the leaf area, crown volume and canopy dynamics, are often not included for individual trees. For realistic predictions of the leaf biomass, variables other than *H* must be included [70–72]. The tree *CR* is a useful indicator of vigor and stand density [73–75]. Measurements of crown dimensions have been recently emphasized as important to improving tree biomass estimation, including measurements of the crown length, the crown width and the diameter of the largest branch in a tree [29, 33]. Wang *et al*. [76] demonstrated that crown width is an important determinant of leaf biomass for Korean pine (*Pinus koraiensis*). We also found that equations with the variable *CR* greatly improved root biomass estimates (Tables 2 and 4). However, some studies reported that root biomass equations were not improved by including crown width [77–78]. In addition, tree age is the other factor. Disregarding tree age may give biased biomass estimates [79]. In this study, we developed a biomass model for each tree component by age group, which could be of benefit for forest managers when evaluating biomass storage and carbon sequestration for *C*. *equisetifolia* in the tropical forest of Hainan Island.

When we estimate biomass from tree components, it is desirable to have the property of additivity in the biomass estimations of the components. The principle of additivity in which the biomass estimations from component equations added to the total biomass has long been recognized [16]. Parresol [48] found that a seemingly unrelated regression (SUR) method can be applied when considering the contemporaneous correlations among different components and biomass additivity, including the trunk, the branch, the leaf, and the root, from the same tree. The SUR method led to efficient parameter estimates by considering inherent correlations among biomass components. Russell [80] reported that the largest gain in using the SUR method is that confidence and prediction intervals for biomass estimates are narrower than isolated estimates. In this study, based on the best individual regressions for each component independently in young, middle-aged and mature forests, the systems of equations presented herein will provide reasonable estimates for those who wish to estimate the biomass of *C*. *equisetifolia* trees in the tropical forest of Hainan Island (Figs 2–7).

## Conclusion

The tree biomass of *C*. *equisetifolia* over the young, middle and mature three age groups in a tropical forest of Hainan Island was highest in the trunk, followed by (in decreasing order) the root, the branch, and the leaf. The biomass from the trunk increased with forest age, while that in the branch and the leaf declined, especially for the leaf. In this study, 12 equations for biomass components by three age groups were established and the best models for estimating tree biomass components were selected according to the R^2^ and MAPE. Among these 12 equations, only three equations with variable *D* were made, while the remaining equations introduced the other variables, including *H*, *D*^2^*H*, and *CR*. An equation including the *CR* greatly improved the model performance and reduced the error, especially for the young and mature forests. Also taking into account the biomass additivity, our findings suggest that the seemingly unrelated regression (SUR) not only gave efficient and accurate estimates but also achieved the logical additivity of biomass for *C*. *equisetifolia* in a tropical forest of Hainan Island.

## Supporting Information

S1 Table Proportion of tree biomass from components in young, middle-aged, and mature forests. (DOC)
