# Supplementary material for: Tree Biomass Allocation and Its Model Additivity for Casuarina equisetifolia in a Tropical Forest of Hainan Island, China
Source: PLoS One. 2016 Mar 22;11(3):e0151858. doi: 10.1371/journal.pone.0151858 (PMC4803337; doi:10.1371/journal.pone.0151858)
Supplement: S1 Table — (DOC) [file pone.0151858.s001.doc]

**S1 Table. Proportion of tree biomass allocated to components in young-aged forest, middle-aged forest, and mature forest.**

| Group | Trunk | Branch | Leaf | Root |
| --- | --- | --- | --- | --- |
| Young | 0.5894 | 0.1120 | 0.1030 | 0.1955 |
| Young | 0.2317 | 0.3527 | 0.2698 | 0.1459 |
| Young | 0.3035 | 0.2765 | 0.2142 | 0.2058 |
| Young | 0.5383 | 0.1055 | 0.1718 | 0.1843 |
| Young | 0.4879 | 0.1100 | 0.1243 | 0.2777 |
| Young | 0.4824 | 0.1316 | 0.1331 | 0.2529 |
| Young | 0.5120 | 0.1044 | 0.0955 | 0.2881 |
| Young | 0.6272 | 0.0882 | 0.1011 | 0.1835 |
| Young | 0.5173 | 0.1424 | 0.1403 | 0.2000 |
| Young | 0.5893 | 0.0943 | 0.1190 | 0.1974 |
| Young | 0.6073 | 0.0841 | 0.1243 | 0.1843 |
| Young | 0.2888 | 0.2701 | 0.2834 | 0.1577 |
| Young | 0.3648 | 0.2146 | 0.2222 | 0.1984 |
| Young | 0.2931 | 0.1439 | 0.3356 | 0.2274 |
| Young | 0.5148 | 0.1351 | 0.1678 | 0.1823 |
| Young | 0.5634 | 0.1366 | 0.0550 | 0.2450 |
| Young | 0.5174 | 0.0916 | 0.1459 | 0.2451 |
| Young | 0.5430 | 0.0916 | 0.1389 | 0.2264 |
| Middle | 0.5657 | 0.0964 | 0.1368 | 0.2012 |
| Middle | 0.6215 | 0.0759 | 0.0768 | 0.2259 |
| Middle | 0.5840 | 0.1154 | 0.0704 | 0.2303 |
| Middle | 0.5695 | 0.0726 | 0.0506 | 0.3073 |
| Middle | 0.5223 | 0.1035 | 0.0895 | 0.2847 |
| Middle | 0.5662 | 0.0906 | 0.0828 | 0.2603 |
| Middle | 0.5596 | 0.0721 | 0.0757 | 0.2926 |
| Middle | 0.5959 | 0.0948 | 0.0749 | 0.2344 |
| Middle | 0.5918 | 0.1073 | 0.0635 | 0.2374 |
| Middle | 0.6201 | 0.1404 | 0.0585 | 0.1811 |
| Middle | 0.6424 | 0.1038 | 0.0623 | 0.1915 |
| Middle | 0.4391 | 0.1852 | 0.1554 | 0.2203 |
| Middle | 0.3396 | 0.2288 | 0.1669 | 0.2646 |
| Middle | 0.5445 | 0.1432 | 0.0993 | 0.2130 |
| Middle | 0.5379 | 0.0964 | 0.0986 | 0.2671 |
| Middle | 0.6942 | 0.0877 | 0.0672 | 0.1509 |
| Middle | 0.7572 | 0.0716 | 0.0466 | 0.1245 |
| Middle | 0.6494 | 0.1278 | 0.0555 | 0.1674 |
| Middle | 0.5665 | 0.2066 | 0.0921 | 0.1348 |
| Mature | 0.5926 | 0.1425 | 0.0609 | 0.2039 |
| Mature | 0.6706 | 0.0695 | 0.0503 | 0.2096 |
| Mature | 0.6069 | 0.1418 | 0.0592 | 0.1921 |
| Mature | 0.6548 | 0.0914 | 0.0582 | 0.1956 |
| Mature | 0.6159 | 0.1113 | 0.0658 | 0.2070 |
| Mature | 0.6714 | 0.0864 | 0.0309 | 0.2113 |
| Mature | 0.6287 | 0.0729 | 0.0420 | 0.2564 |
| Mature | 0.4649 | 0.1388 | 0.1370 | 0.2593 |
| Mature | 0.5743 | 0.1030 | 0.0718 | 0.2509 |
| Mature | 0.6373 | 0.0905 | 0.0299 | 0.2423 |
| Mature | 0.6294 | 0.0876 | 0.0347 | 0.2483 |
| Mature | 0.6567 | 0.0655 | 0.0405 | 0.2373 |
| Mature | 0.6882 | 0.0605 | 0.0591 | 0.1923 |
| Mature | 0.7182 | 0.0506 | 0.0459 | 0.1853 |
| Mature | 0.6256 | 0.1057 | 0.0954 | 0.1733 |
| Mature | 0.5656 | 0.1328 | 0.0527 | 0.2489 |
| Mature | 0.4932 | 0.1340 | 0.1289 | 0.2439 |
| Mature | 0.6777 | 0.0807 | 0.0439 | 0.1978 |
| Mature | 0.6286 | 0.1369 | 0.0580 | 0.1765 |
| Mature | 0.7229 | 0.0394 | 0.0181 | 0.2196 |
| Mature | 0.6911 | 0.0533 | 0.0512 | 0.2043 |
| Mature | 0.6820 | 0.0862 | 0.0355 | 0.1963 |
| Mature | 0.6545 | 0.0676 | 0.0676 | 0.2103 |
| Mature | 0.6040 | 0.0475 | 0.0648 | 0.2837 |
| Mature | 0.5759 | 0.1505 | 0.0801 | 0.1935 |
| Mature | 0.5676 | 0.0611 | 0.0860 | 0.2852 |
| Mature | 0.5905 | 0.0656 | 0.0657 | 0.2782 |
| Mature | 0.4770 | 0.1884 | 0.1437 | 0.1908 |
| Mature | 0.6849 | 0.0524 | 0.0472 | 0.2156 |
| Mature | 0.6881 | 0.0490 | 0.0529 | 0.2100 |
| Mature | 0.7090 | 0.0479 | 0.0519 | 0.1912 |
| Mature | 0.6270 | 0.1062 | 0.0515 | 0.2154 |
| Mature | 0.6722 | 0.0987 | 0.0357 | 0.1934 |
| Mature | 0.6334 | 0.0731 | 0.0771 | 0.2164 |
| Mature | 0.6174 | 0.1397 | 0.0725 | 0.1704 |
